
JOURNAL INFORMATION
==============================
Journal ID: Wirtschaftsdienst

Wirtschaftsdienst

EISSN: 1613-978X

ARTICLE INFORMATION
==============================
PMCID: PMC9307431
DOI: 10.1007/s10273-022-3232-2
Article ID: 3232
Article version: 1
Subjects: Ökonomische Trends

Rückkehr zu stärkerem Beschäftigungswachstum in den Städten erwartet

Return to Stronger Employment Growth Expected in Cities

Rossen Anja Anja.Rossen4@iab.de

grid.425330.3 0000 0001 1931 2061 der Bundesagentur für Arbeit (BA), Institut für Arbeitsmarkt- und Berufsforschung, Regensburger Straße 104, 90478 Nürnberg, Deutschland
Electronic publication date: 2022 Jul 23
Print publication date: 2022
Volume: 102
Issue: 7
First page: 568
Last page: 570
Copyright: © Der/die Autor:in 2022, korrigierte Publikation 2022
License: Open Access: Dieser Artikel wird unter der Creative Commons Namensnennung 4.0 International Lizenz veröffentlicht (https://creativecommons.org/licenses/by/4.0/deed.de).
Open Access wird durch die ZBW — Leibniz-Informationszentrum Wirtschaft gefördert.
License URL: https://creativecommons.org/licenses/by/4.0/

Keywords: C53, R23
issue-copyright-statement: © The Author(s) 2022

==============================

Das Institut für Arbeitsmarkt- und Berufsforschung (IAB) erstellt zweimal jährlich Prognosen über die Entwicklung verschiedener Arbeitsmarktgrößen, darunter die Zahl der Beschäftigten und der Arbeitslosen sowie deren Veränderungsrate im Vergleich zum Jahresdurchschnitt des Vorjahrs. Neben der Prognose für Deutschland (Gartner et al., 2022) werden dabei auch Prognosen für verschiedene regionale Einheiten vorgenommen: für Ost- und Westdeutschland, die Bundesländer sowie die Agenturbezirke der Bundesagentur für Arbeit (siehe Heining et al., 2022a für methodische Hinweise). Das IAB bedient damit den Bedarf verschiedener Arbeitsmarktakteure nach einer wissenschaftlich fundierten Einschätzung zur weiteren Entwicklung des Arbeitsmarkts auf bundesweiter wie auf regionaler Ebene.

In diesem Beitrag wird eine neue Facette der IAB-Regionalprognosen vorgestellt. Für die Entwicklung im Jahr 2022 berichten wir Ergebnisse auf der Ebene der siedlungsstrukturellen Kreistypen des Bundesinstituts für Bau-, Stadt- und Raumforschung (BBSR). Die BBSR-Klassifizierung beruht auf den 361 Kreisregionen, die gemäß ihrer Bevölkerungsgröße und -dichte (insgesamt sowie den in diesen Regionen enthaltenen Groß- und Mittelstädten) in vier Gruppen eingeteilt werden: kreisfreie Großstädte, städtische Kreise, ländliche Kreise mit Verdichtungsansätzen sowie dünn besiedelte ländliche Kreise. Die räumliche Verteilung der siedlungsstrukturellen Kreistypen wird dabei nicht bereits von den administrativen Einheiten bestimmt. So gibt es beispielsweise kreisfreie Großstädte mit Ausnahme des Saarlands in jedem Bundesland. Der Großteil der Kreisregionen Ostdeutschlands fällt in die Kategorie der beiden ländlichen Kreistypen. Solche finden sich aber auch häufig in Bayern, Niedersachsen, Rheinland-Pfalz und Schleswig-Holstein.

Die Beschäftigung ist nicht einheitlich über die vier Kreistypen verteilt. Im Jahr 2021 war die jahresdurchschnittliche Beschäftigung in kreisfreien Großstädten mit etwa 12.678.000 Beschäftigten am höchsten, gefolgt von den städtischen Kreisen (11.756.000). Die Beschäftigung in den beiden ländlichen Regionstypen lag dagegen mit 5.088.000 (ländliche Kreistypen mit Verdichtungsansätzen) und 4.385.000 (dünn besiedelte Kreise) deutlich niedriger. Die siedlungsstrukturellen Kreistypen unterscheiden sich auch hinsichtlich ihrer Arbeitsmarktentwicklung. So betrug die Wachstumsrate der Beschäftigung im Durchschnitt der Jahre von 2000 bis 2021 in den kreisfreien Großstädten etwa 1,1 %. Damit war sie höher als in den anderen drei Kreistypen und fast doppelt so hoch wie in den dünn besiedelten Kreisen (0,6 %). Eine Differenzierung nach Strukturtypen ist auch mit Blick auf die COVID-19-Pandemie von Interesse. Die Pandemie stellt eine deutliche Zäsur der Arbeitsmarktentwicklung im Vergleich zu den Vorjahren dar, die zudem regional unterschiedlich ausgefallen ist (Hamann et al., 2021; Buch et al., 2022). Vor diesem Hintergrund stellt sich die Frage, ob es eine Rückkehr zu den vor der Pandemie bestehenden Unterschieden in der regionalen Arbeitsmarktentwicklung geben wird oder ob sich nachhaltige Veränderungen einstellen. Da die COVID-19-Pandemie weiter anhält, kann eine Prognose für das Jahr 2022 diese Frage zwar nicht abschließend beantworten, erlaubt aber dennoch eine erste Einschätzung.

Das IAB rechnet in seiner Bundesprognose mit einem Anstieg des Bruttoinlandprodukts im Jahr 2022 um etwa 1,5 % (Gartner et al., 2022). Der Arbeitsmarkt hat sich bislang recht robust entwickelt, was das IAB tendenziell auch für das laufende Jahr erwartet. Nach einem sehr starken Anstieg der Arbeitslosigkeit im Jahr 2020 zeigte sich 2021 im Zuge der moderaten konjunkturellen Erholung bereits ein Abbau der Arbeitslosigkeit. Dieser wird sich fortsetzen, sodass die Zahl der Arbeitslosen in Deutschland im Jahr 2022 im Jahresdurchschnitt voraussichtlich um 351.000 Personen bzw. 13,4 % auf 2,3 Mio. zurückgehen wird. Für die sozialversicherungspflichtige Beschäftigung wird für 2022 eine Zunahme um 521.000 Personen bzw.

Abbildung 1
**Wachstumsrate der Beschäftigung**
in %

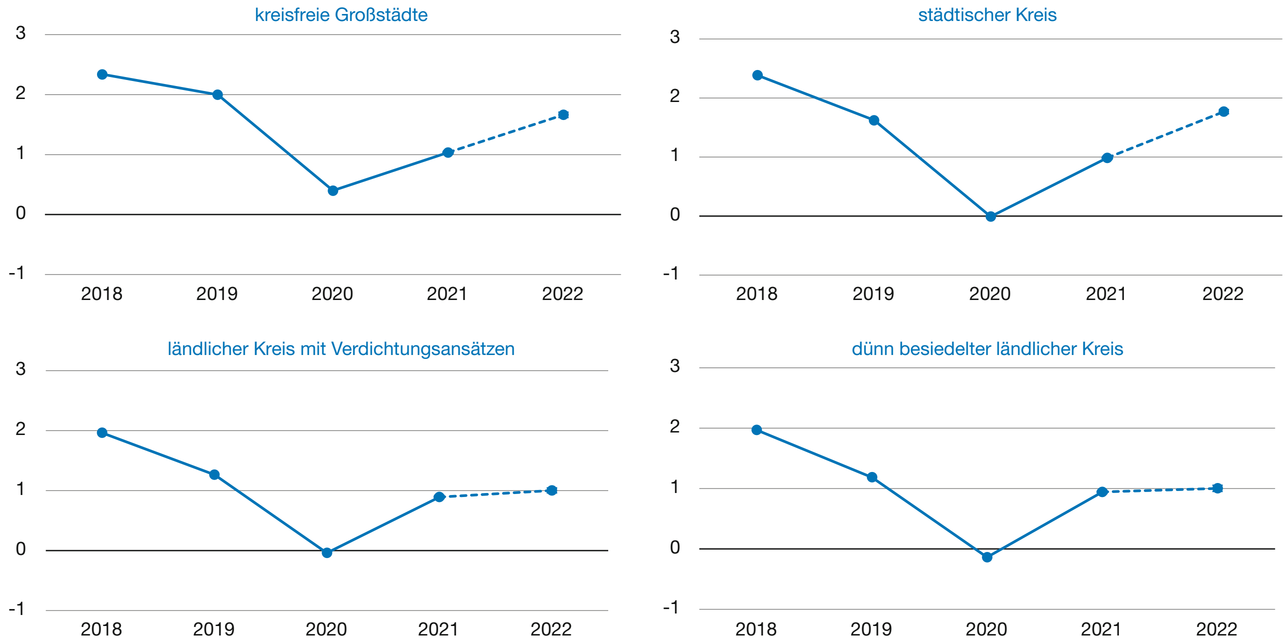

Quelle: IAB, BBSR, eigene Berechnungen.

1,5 % auf 34,4 Mio. erwartet. Auf regionaler Ebene geht das IAB von einem etwas höheren Beschäftigungsanstieg und einem etwas kräftigeren Rückgang der Arbeitslosigkeit in Westdeutschland als in Ostdeutschland aus (Heining et al., 2022b). Angesichts des Kriegs in der Ukraine, der Lieferengpässe sowie der anhaltenden COVID-19-Pandemie sind diese Prognosen jedoch mit großer Unsicherheit behaftet.

Abbildung 1 zeigt die Veränderungsrate der jahresdurchschnittlichen Beschäftigung für die vier Siedlungsstrukturtypen des BBSR. Die Abbildung veranschaulicht, dass sich die Beschäftigung vor Beginn der Pandemie nicht einheitlich entwickelt hat, sondern das Wachstum in den kreisfreien Städten und den städtischen Kreisen höher lag als in den beiden ländlichen Strukturtypen. Deutlich zeigt sich für die Wachstumsrate des Jahres 2019, die in den ländlichen Strukturtypen mit rund 1 % etwa halb so hoch lag wie in den kreisfreien Großstädten. Im Verlauf der COVID-19-Pandemie kam es im Jahr 2020 zunächst über alle Strukturtypen hinweg zu einem starken Einbruch des Beschäftigungswachstums (das aber in den kreisfreien Großstädten mit 0,5 % im Jahr 2020 etwas höher war als in den anderen Typen), dem eine ähnlich ausgeprägte Erholung im Jahr 2021 mit Wachstumsraten von knapp 1 % in allen vier Strukturtypen folgte. Für das Jahr 2022 erwarten wir dagegen wieder Unterschiede in der Beschäftigungsentwicklung. Während wir in den städtischen Strukturtypen von einem Anstieg des Wachstums auf knapp unter 2 % ausgehen, rechnen wir in den beiden ländlichen Strukturtypen mit Stagnation.

Anders fallen dagegen die regionalen Unterschiede bei der Arbeitslosigkeit aus. Wie in Abbildung 2 dargestellt, sind die Veränderungen der Arbeitslosigkeit in den beiden ländlichen Strukturtypen im Durchschnitt günstiger als in den städtischen Räumen. Vor Beginn der Pandemie ist es 2018 in den ländlichen Räumen zu einem Rückgang der Arbeitslosigkeit um rund 10 % gekommen, während die entsprechende Veränderungsrate in den beiden städtischen Regionen absolut gesehen etwas niedriger war. Der Abbau der Arbeitslosigkeit setzte sich im Folgejahr bereits nur in abgeschwächter Form fort, wobei die Zahl der Arbeitslosen in den kreisfreien Städten und den städtischen Kreisen nahezu stagnierte. Deutlich zu erkennen ist der Anstieg der Arbeitslosigkeit im ersten Jahr der COVID-19-Pandemie. Gegenüber dem Vorjahr stieg die Zahl der Arbeitslosen in den städtischen Regionen um rund 20 %, wohingegen der Anstieg auf dem Land etwas geringer ausfiel. Während die Arbeitslosigkeit in den kreisfreien Städten 2021 noch auf hohem Niveau stagnierte, stellte sich in den drei anderen Strukturtypen bereits eine leichte Erholung ein. Für das Jahr 2022 gehen wir von einem ähnlich hohen Rückgang in der Arbeitslosigkeit von rund 13 % aus. Damit würde die Veränderung in der Wachstumsrate vor allem in den kreisfreien Städten relativ hoch

Abbildung 2
**Wachstumsrate der Arbeitslosenzahl**
in %

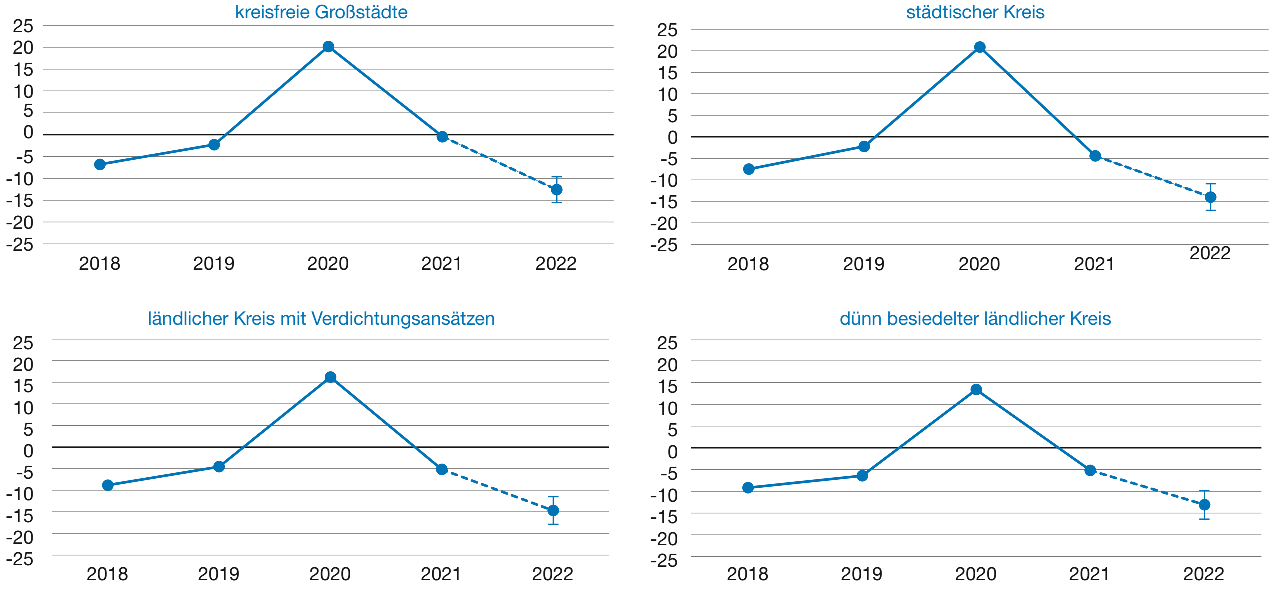

Quelle: IAB, BBSR, eigene Berechnungen.

ausfallen und der zunächst ausgebliebene Abbau der Arbeitslosigkeit zum Teil nachgeholt werden.

Insgesamt legen die Prognosen für das Jahr 2022 eine Rückkehr zu den schon vor der Pandemie bestehenden regionalen Disparitäten mit Blick auf die Entwicklung von Arbeitslosigkeit und Beschäftigung nahe. Während sich bei der Prognose der Arbeitslosigkeit keine größeren Unterschiede zwischen den Strukturtypen zeigen, nimmt das erwartete Beschäftigungswachstum in den kreisfreien Großstädten und den städtischen Kreisen gegenüber dem Vorjahr zu, stagniert aber im Fall der beiden ländlichen Strukturtypen. Ob es sich dabei um eine Rückkehr zu den bereits vor der Pandemie bestehenden Unterschieden handelt oder sich die regionalen Disparitäten womöglich noch verschärfen, wird sich zeigen.

This paper presents a new facet of the regional forecasts of the Institute for Employment Research (IAB). For the development in 2022, we report results at the level of administrative district types of the Federal Institute for Research on Building, Urban Affairs and Spatial Development (BBSR). Overall, the forecasts for 2022 suggest a return to the regional disparities that already existed before the pandemic with regard to the development of unemployment and employment. While there are no major differences between the administrative district types in the unemployment forecast, the expected growth in employment increases in cities and urban areas compared to the previous year, but stagnates in the case of the two rural structural types.

Dr. Anja Rossen ist wissenschaftliche Mitarbeiterin im Regionalen Forschungsnetz des Instituts für Arbeitsmarkt- und Berufsforschung (IAB) in Nürnberg.

The original online version of this article was revised due to incorrect page number.

Change history

9/12/2022

Zu diesem Beitrag wurde ein Erratum veröffentlicht: 10.1007/s10273-022-3263-8


Literatur

Buch T Eigenhüller L Hamann S Kropp P Otto A Roth D Seibert H Regionale Arbeitsmarktdisparitäten in der ersten Corona-Welle WSI-Mitteilungen 2022 75 2 96 106
Gartner, H., T. Hellwagner, M. Hummel, C. Hutter, S. Wanger, E. Weber und G. Zika (2022), IAB-Prognose 2022: Konjunkturaufschwung ausgebremst, IAB-Kurzbericht, 7.
Hamann, S., P. Kropp, A. Niebuhr, D. Roth und G. Sieglen (2021), Die regionalen Arbeitsmarkteffekte der Covid-19-Pandemie: Nicht nur eine Frage der Wirtschaftsstruktur, IAB-Kurzbericht, 14.
Heining, J., O. Jost, A. Rossen, D. Roth und A. Weyh (2022a), Regionale Arbeitsmarktprognosen, 1, aktuelle Daten und Indikatoren.
Heining, J., O. Jost, A. Rossen, D. Roth, C. Teichert und A. Weyh (2022b), Regionale Arbeitsmarktprognosen (Stand: März 2022), IAB-Forum, 4. April 2022, https://www.iab-forum.de/regionale-arbeitsmarktprognosen-stand-maerz-2022/ (19. Mai 2022).
